# LiDAR-Based Real-Time Detection and Modeling of Power Lines for Unmanned Aerial Vehicles

**DOI:** 10.3390/s19081812

**Published:** 2019-04-16

**Authors:** Fábio Azevedo, André Dias, José Almeida, Alexandre Oliveira, André Ferreira, Tiago Santos, Alfredo Martins, Eduardo Silva

**Affiliations:** 1 INESC Technology and Science, Centre for Robotics and Autonomous Systems, 4200-465 Porto, Portugal; 2 ISEP-School of Engineering, Electrical Engineering Department, 4200-072 Porto, Portugal

**Keywords:** power line, Light Detection And Ranging (LiDAR), real-time, Unmanned Aerial Vehicle (UAV), point cloud, segmentation, catenary

## Abstract

The effective monitoring and maintenance of power lines are becoming increasingly important due to a global growing dependence on electricity. The costs and risks associated with the traditional foot patrol and helicopter-based inspections can be reduced by using UAVs with the appropriate sensors. However, this implies developing algorithms to make the power line inspection process reliable and autonomous. In order to overcome the limitations of visual methods in the presence of poor light and noisy backgrounds, we propose to address the problem of power line detection and modeling based on LiDAR. The PL${}^{2}$DM, Power Line LiDAR-based Detection and Modeling, is a novel approach to detect power lines. Its basis is a scan-by-scan adaptive neighbor minimalist comparison for all the points in a point cloud. The power line final model is obtained by matching and grouping several line segments, using their collinearity properties. Horizontally, the power lines are modeled as a straight line, and vertically as a catenary curve. Using a real dataset, the algorithm showed promising results both in terms of outputs and processing time, adding real-time object-based perception capabilities for other layers of processing.

## 1. Introduction

During the last few years, there has been an increase in the research effort in the field of aerial robotics, leading to a growth of the application scenarios with UAVs [1]. Furthermore, this wider set of applications is related to a continuous change on the research focus, which is starting to focus on higher level tasks (such as navigation and task planning, paying attention to visual odometry, localization and mapping). Until the beginning of this century, the UAV research was mainly focused on hardware development, modeling and control. In the most recent years, researchers are giving more importance to topics related to obstacle detection and collision avoidance. Nowadays, UAVs can be applied for search and rescue missions, surveillance and inspection of structures, among others [2,3]. Due to their ability of collecting data from different positions, angles and distances, and supporting a reasonable payload of sensors, multirotor UAVs are suitable for the inspection of electric assets, such as electric pylons and insulators, being, therefore, interesting for electrical power providers [4].

Due to the growing dependence of many modern-day societies on electricity, there is an increasing importance of effective monitoring and maintenance of power lines [5], as the distribution of the power from the source to the user is one of the key factors in the quality of the service provided by the electrical power companies. Considering the progressive development of new green power generation plants in Europe, the need for a higher and more reliable electrical transport capacity is growing, however, the installation of new electric power lines is usually not accepted by the population [6]. This leads to a constant operation at the maximum capacity of the power lines, without having redundancies or reserves to compensate breakdowns. In order to avoid possible economic losses and blackouts for the consumers, the electrical power companies need to adopt a preventive and predictive maintenance philosophy by means of, for example, a periodic visual and thermal inspection [6,7].

Power line inspection takes into account not only their elements but also the surrounding objects, especially vegetation [6,7,8]. When the minimum clearance between the vegetation and the conductors or assets is violated, tree falls or conductors oscillation during bad weather conditions can lead to the short-circuiting of the line, causing widespread outages [9,10,11] or even bush-fires, especially in drier environments [7,8,12,13,14]. These threats result in a general acceptance on considering vegetation as one of the most hazardous factors for the overhead power lines’ integrity [8,9,10,11,12].

The inspections of the power lines traditionally rely on human labor [6,7,8,12], as they are primarily performed on foot by a team driving between spans or from a helicopter flying alongside the line. The power structures can be installed on complex and harsh environments, which makes the on-foot patrols time-consuming and poorly efficient. Helicopter-based inspections are more time-efficient but are both expensive and taxing on the pilot and operator [12,15,16].

A proper inspection of the power line elements can be done by using laser scanning data, optical [17] and thermal [18,19] images obtained by UAVs [6,15,20,21]. Due to their high 3D maneuverability, the multirotor UAVs are one of the most suitable vehicles for a complete inspection of the power line elements. Despite the advantages, using a multirotor UAV in a complex and confined environment can be challenging for the pilot and the autonomous mission planning. A noisy background can make the conductors visually undetectable by the pilot [15]. This raises the need for an autonomous module of obstacle detection and collision avoidance that is capable of actuating in both autonomous and assisted modes [22].

This paper addresses the development of an algorithm for real-time power line detection using LiDAR high-rate data, the Power Line LiDAR-based Detection and Modeling (PL${}^{2}$DM). There is a lack of studies in the literature that consider (simultaneously) the power line detection, the large amount of data and the real-time operation. The main objective of this work is to develop a methodology that contributes to an object-based perception for a UAV. Its main focus is the detection of power line conductors and the prediction of their extension, through the estimation of their mathematical model. The PL${}^{2}$DM is a scan-based algorithm that segments a point cloud, by a minimalist range-based neighbor comparison using planar analysis, and extracts a set of power line candidate points. Those points are fitted to line segments that are further matched and properly grouped, based in their collinearity properties. The final estimated mathematical model of a power line is given by a horizontal straight line combined with a vertical catenary curve.

The paper is outlined as follows: Section 2 presents a preliminary study of the works related with the point cloud segmentation and power lines extraction. The Section 3 contains an overview of the hardware and software required to implement the algorithm, followed by its description in Section 4. After that, in Section 5, we discuss the obtained results, with the conclusions and suggestions for future work placed in the Section 6.

## 2. Related Work

LiDAR is an active remote sensor that uses a laser in the visible or near-visible part of the electromagnetic spectrum to obtain measures [23]. Scientists have used this kind of sensor since the 1960s [23,24]. In the early 1970s there were some of the first space-based LiDAR measurements from lunar orbit using the laser altimeter on the Apollo 15 mission [25]. Since the lasers became widely available, ground-based and airborne LiDAR systems started to increase steadily [23].

### 2.1. Segmentation

The extraction of valuable spatial data from the large amount of information that LiDAR sensors can provide is difficult and time-consuming; therefore, segmentation is generally a prerequisite for feature extraction.

The first segmentation techniques relied on 2.5D grid or image data [26,27], interpolating the point cloud for applying some image-based segmentation and classification, however, some important spatial information could be lost [28,29]. To overcome the loss of spatial information, Wang and Tseng [30,31] proposed an octree-structure-based split-and-merge segmentation method more suitable for LiDAR data.

For urban environments, Shan and Sampath [32] presented a binary segmentation, labeling each point as either ground or non-ground. Using a ground vehicle, Steinhauser et al. [33] analyzed the 2D scan from each angular step of the LiDAR to find drivable road. A line fitting is then performed to mark the points as obstacle, surface or critical surface. In [34], Moosmann et al. used a local convexity criteria for dealing with non-flat grounds. Using ordered points, it is estimated a local surface normal, as in [35], and the segmentation is based on the comparison of two neighbor surfaces.

Klasing et al. developed a segmentation based on the Radially Bounded Nearest Neighbors (RBNN) graph [36]. Each node is connected to all neighbors that lie within a predefined radius, ignoring all clusters with less than a defined minimum number of points. The refined version of the algorithm [37] has real-time capabilities. It continuously monitors the Nearest Neighbors (NNs) and uses a feature space consisting on both ordered incoming points and their estimated normal vectors for clustering.

In [38], the authors generated a 2.5D ego-centered occupancy grid, for each LiDAR revolution, and stored the maximum absolute *z* difference of all points in a cell, like in [39]. The 3D points are then recovered and the object classification is made using point feature histograms and a previously trained Support Vector Machine (SVM). For preventing the under-segmentation, Himmelsbach et al. [40] improved the method by organizing the points in both direction and range. Getting the point with the lowest height for each bin of the discretized range, it applies an Incremental Algorithm [41] for line fitting and ground plane detection. In terms of runtime values, this method has outperformed the RBNN [36].

In [42] is shown the benefit of ground extraction prior to object segmentation for dense data. The *Cluster-All with Variable Neighborhood* was the method that had the best trade-off in terms of simplicity, accuracy and computational times. For sparse data, both the Gaussian Process INcremental SAmple Consensus (GP-INSAC) and the mesh-based techniques provided close to real-time performance. In [43], the authors made a comparison between the use of rectangular and radial grids centered on the sensor. In this analysis, the radial grid requires less cells and produces less fragmentation of the objects, when compared to the rectangular, which leads to a faster processing. The core of this approach is the use of smaller images instead of large point cloud data for processing.

In 2012, Choe et al. [44] proposed an improvement of the RBNN algorithm, like in [37], for urban environments. All the points that lie on horizontal surfaces are potential ground points and are removed from further calculations if lie on the larger horizontal cluster. The remaining points are then clustered regarding not a fixed [37] but a distance-varying radius. For non-horizontal points, the segmentation in [45] is based on an Ellipsoid model based RBNN (e-RBNN), that uses an ellipsoid rather than a circular region, defining a scaled radius based on the neighbor points distance, and another based on the sensor-to-point Euclidean Distance (ED).

Reddy and Pal [46] presented a region-growing-based algorithm. The segmentation is dependent on the unevenness value that is calculated based on the expected and measured ranges difference. Thus, this method relies on the knowledge of the sensor’s scan geometry. Later, in 2017, there was an approach of 3D segmentation more directed to autonomous ground vehicles [47]. It uses as input a 360-degree coverage point cloud and performs a two-step segmentation: the ground is extracted based on a Ground Plane Fitting (GPF) followed by a clustering methodology named Scan Line Run (SLR).

Other existing works also use image color information from a camera [48] or Extruded Surface of Cross Sections (ESCSs) [49] for segmenting 3D point clouds.

### 2.2. Line Extraction

The use of LiDAR sensors to provide a 3D model of the power lines is useful, both to evaluate their mechanical conditions and to detect and monitor some possible dangers, like encroaching objects [9,13,17,50]. This modeling can provide a multifaceted analysis of power line maintenance and risk evaluation, saving time and costs [51].

Melzer and Briese [52] introduced a method to reconstruct power lines using an airborne LiDAR. After filtering the terrain and vegetation, an iterative version of Hough Transform (HT) [53] is applied to extract lines on the horizontally projected points. After grouping the detected lines, their model is fitted to a catenary with parameters obtained from randomly selected line primitives based on RANdom SAmple Consensus (RANSAC) [54]. In [55], the authors presented a stochastic method for separating trees and power lines in an urban environment. The point cloud is preprocessed to extract buildings.

In [56,57], the authors presented a technique for clearance anomalies detection. It starts to consider real-time operation using a sweeping 2D LiDAR attached to the helicopter fuselage, acquiring at a rate of 2 Hz. Using global positioning, the power lines are modeled by a polynomial fitting, as in [58], which allows to recover the line detection when some gaps occur.

McLaughlin, in [59], proposes a helicopter point cloud segmentation method for power lines extraction. Using an ellipsoid-based neighborhood search along the direction of flight, it classifies each cluster based on the eigenvalues of its covariance matrix. Each possible power line cluster is then specified by its mean point and largest eigenvalue. The line span is extracted by fitting the power line to a straight line, on the horizontal plane, and a catenary, on the vertical plane, along that direction.

The work of Jwa et al. [60] presents a voxel segmentation technique to classify voxels as linear, non-linear or undefined. This classification is performed by meeting trained parameters of the HT, eigenvalues, and point density combination. To have an initial orientation, for each power line candidate point is performed an eight-hypothesis Compass Line Filter (CLF). The proposed Voxel-based Piece-wise Line Detector (VPLD) process will propagate the sub-cubic boxes based on the previous information of the estimated catenary model. If the number of points is not enough to model a catenary, a line equation is used instead.

In 2014, Xiang [61] approached the importance of a proper filtering of data in the quality of the final results. The power lines are fitted and reconstructed by using a line model for the horizontal plane, and a catenary for the vertical one. The hazard tree detection is also discussed, however, only vertical hazards are effectively detected. In [62], the initial point cloud is voxelized and filtered according to terrain clearance, up-down continuity, and feature eigenvector. A HT is applied to the horizontally projected points for detecting the power lines. A cluster-growing method based on local straight line fitting is used to reach the final fitting to a polynomial.

In [63], the authors proposed a method for ground vehicles. It extracts off-road points, segmenting them using height, spatial density, and size and shape filters. The power line candidate points are extracted using HT and then clustered based on their distance. The mathematical fitting of the power lines to a line on the horizontal and a catenary on the vertical plane can then be used to aid the pylon detection. Another method for pylon detection is presented in [64] for aerial vehicles.

Guo et al. [65] prevented the false-positive classification of vegetation as pylons by verifying if the pylons are connected by wires, using a HT. The power line points that belong to a span between two consecutive pylons are segmented into profiles orthogonal to the span direction and their similarity is checked. Using a RANSAC-like method for setting the initial parameters of the catenary curve, the span is iteratively reconstructed and the points are added to the estimation if they satisfy the so far predicted model.

An automatic clearance anomaly detection algorithm was developed in [8]. It performs a span-by-span analysis, segmenting the power line points into clusters. The resulting clusters are fitted into a horizontal linear and a vertical catenary model.

Regarding the power line model fitting, Jaw et al. in [11] studied the effect of the wind on the conductors’ motion while they are acquired by LiDAR sensors.

### 2.3. Overall Analysis

Excluding the direction of the span, the power lines have a reduced size, which makes them hard to detected continuously by a LiDAR. Because of that, all the data available is of great potential interest for analysis. However, the algorithm aims to process the data in the minimum time possible, in order to be able to detect the power lines in real-time and serve as input for obstacle detection algorithms. Due to the inter-dependency between the amount of data and the processing time, the environment discretization [30,31,43] and the 3D to 2D projections (2.5D grids) [26,27,38,39,40] are undesirable. In a grid-like map, it is possible that the power lines points become isolated in a cell, which difficults their evaluation. Adding to that, the projection of points may cause the loss of spatial information. Even if they are then recovered [38], there is an overhead in the processing time, associated with that process.

Several works have shown the benefit of the ground extraction before the data analysis [42,44,47]. The advantage of this technique is being able to ignore the cluster with the most points and, therefore, greatly reduce the amount of data to analyze. For ground vehicles, this assumption is almost always true, however, it might fail for UAVs. As the aerial vehicle’s operation is independent on the ground nature and can be done in both urban and rural environments, the ground may have a lot of variations and be similar to, for example, the top of buildings. One challenger is the fact that the aerial vehicles can easily change its altitude, changing the point density of the detected structures below them, which leads to situations where the ground might not be the cluster with more points.

In what concerns the processing time, the existing works are consistent into directly or indirectly showing the importance of knowing the LiDAR sensor data structure for organizing the incoming points [34,37,40,46]. This prior information vastly decreases the number of operations needed, due to the knowledge of the points’ neighborhood, as clearly evidenced by the transition from the work [36] to [37] of Klasing et al. Given the radial nature of most LiDARs, the data becomes sparser with the distance to the sensor, so the segmentation constrains shall be relaxed for points farther the sensor to prevent over-segmentation [42,43]. Knowing the mechanical structure of the LiDAR can also help to set segmentation constraints [46] based in the expected range difference between neighbor beams. Associating the images of a camera [48] adds information for the segmentation, but it may not be worthwhile due to the processing time increase.

Most of the works more related with the power line extraction have as input a complete point cloud from a dataset, not being, therefore, real-time solutions. The HT is mostly used to detect power lines into horizontally projected points [52,60,62,63,65]. Adding to the disadvantages mentioned above regarding the 2D projections, the HT is also sensitive to noise, which can compromise the results.

Although it is only suitable for small amounts of data, Vale and Gomes-Mota [56,57] used a 2D sweeping LiDAR, acquiring at a low rate, to propose a scan-by-scan real-time method. In [59], the authors used a complete point cloud but the analysis is divided into clusters, presenting a method that allows the calculation of the catenary curve parameters. By definition, a catenary curve is a curve formed by a wire, rope, or chain hanging freely from two points that are not in the same vertical line, which is the case of the power lines. Therefore, this work has the main objective of contributing to fill the gap of power line detection in real-time, using a methodology capable of dealing with large amounts of data and produce mathematical models.

## 3. System Design

This section contains an overview of the hardware and software architectures defined for applying the developed algorithm. Taking advantage on the Robotic Operating System (ROS) modular structure, the system’s software is based on this framework, in order to use available ROS packages and favor its integration in other possible future applications.

### 3.1. Hardware

**Figure 1:**
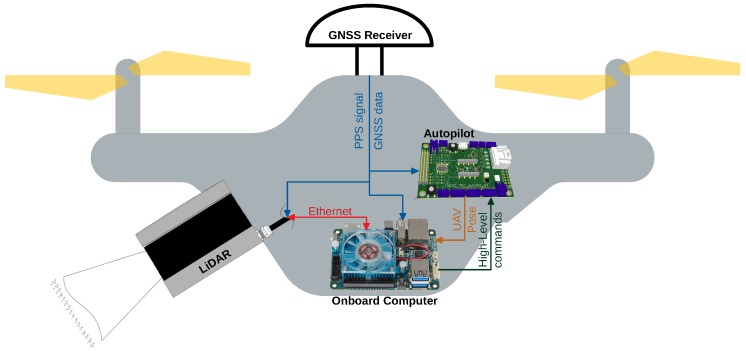
High-level hardware architecture.

The developed algorithm is intended to be applied in a multirotor UAV, using a LiDAR to provide an input 3D point cloud, during an electrical power assets inspection. The onboard computer is responsible for the algorithm processing, using an architecture as the one presented in Figure 1.

Given the large amount of data provided, in the UAV system, the LiDAR sensor needs to establish a communication with the onboard computer through a connection that allows a high transmission rate (like Ethernet in Figure 1, in red). The computer first configures the sensor and then processes all the incoming stream of data.

The Global Navigation Satellite System (GNSS) receiver is responsible for providing data for estimating the UAV pose, by fusing it with the Inertial Measurement Unit (IMU) measurements. Adding to that, the Pulse Per Second (PPS) signal is used to synchronize all the subsystems’ data timestamps.

### 3.2. Software

**Figure 2:**
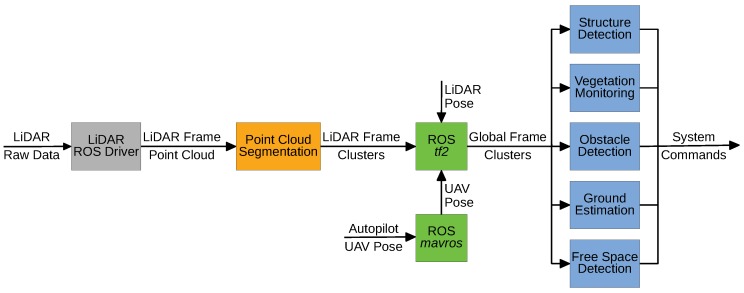
High-level software pipeline.

The main objective of point cloud segmentation is to provide an easier perception of the surrounding environment by creating clusters and detect features using application-specific algorithms. In Figure 2, we expose the high-level software pipeline to segment a point cloud generated by a LiDAR sensor. Apart from the input data, all the pipeline components were developed within the ROS framework.

The estimated UAV pose is provided to the computer (Figure 1, in orange) by means of a topic published by the *mavros* (http://wiki.ros.org/mavros) ROS package. This topic can be used to establish relations between the sensor, UAV, and global coordinate frames using the *tf2* (http://wiki.ros.org/tf2) ROS package, that tracks the available frames’ relations over the time. The LiDAR pose can be either a defined fixed relation with respect to the UAV, if it is fixed to the platform, or a dynamic relation, if attached to a gimbal, for example. In the last case, the LiDAR pose can be defined in a similar way as the UAV’s.

Analyzing Section 2, it is noticeable that the point cloud segmentation can be done at different levels, depending on the desired resolution and features, and the available time. Achieving faster processing requires the knowledge of the LiDAR sensor structure. On 3D spinning LiDARs, due to the large amount of data provided, the azimuth angular resolution is usually high. Therefore, even when attached to a mobile frame, the pose difference is neglectable between two consecutive azimuth measurements, which allows to directly establish comparisons between them, not needing to map it to the global frame. This strategy allows processing time saving and position error reduction, as the UAV pose estimation (and associated errors) is not used.

Using the obtained clusters properly referenced into a global frame, other algorithms, like obstacle or free space detection, can be applied. The detection of obstacles is important to avoid collisions, but the free space recognition is also necessary for navigation and to identify unknown areas. Knowing those areas may be needed for inspection or mapping surveys. Structure detection or vegetation monitoring are application-specific algorithms. For power lines inspection, the classification of the objects into power lines, pylons and vegetation may be necessary to detect faults and potential hazards. All of those algorithms can feed a control system of the UAV either by sending high-level commands to the autopilot (Figure 1, in dark green) or controlling actuators, like releasing some object near to a structure.

## 4. Power Line LiDAR-based Detection and Modeling (PL${}^{2}$DM) Algorithm

Based in the works related to the power line detection and modeling using LiDAR, there is a lack in providing real-time operation for a large amount of data at a high rate. The PL${}^{2}$DM algorithm contributes for overcoming that gap, constituting a real-time solution that is capable of segmenting a point cloud, detect power lines and generate their mathematical model.

**Figure 3:**
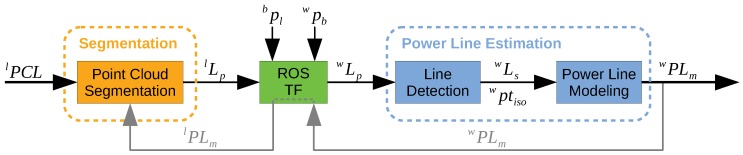
Concept of the proposed algorithm architecture.

The concept of the PL${}^{2}$DM architecture is summarized in Figure 3. Whenever a LiDAR point cloud (${}^{l}PCL$) is available, the segmentation layer is triggered. If there exists any evidence of the power lines existence, the candidate line points (${}^{l}L_{p}$) are provided to the next layer.

The points ${}^{l}L_{p}$ are mapped from the local LiDAR frame (*l*) into the global frame (*w*), using the frame relations. These relations are stored together with the information about the pose of the robot in the global frame (${}^{w}p_{b}$) and the pose of the LiDAR sensor in the robot body (${}^{b}p_{l}$).

The line detection is then performed over the candidate points mapped into the global frame (${}^{w}L_{p}$). The clusters that comprise the collinearity constraints (${}^{w}L_{s}$), or with isolated points (${}^{w}pt_{iso}$), are passed to the power line modeling module. The proposed method tries to match the new information with the one previously stored, trying to model the detected power lines (${}^{w}PL_{m}$).

The information of the obtained models can then be used to feed other algorithms. Moreover, mapping those models into the local frame (${}^{l}PL_{m}$) will support the next segmentation procedures, as they may allow to improve the classification of the clusters.

### 4.1. Segmentation

The segmentation of a point cloud is the first step of the PL${}^{2}$DM. Being the closest layer to the sensor data input, a correct definition of the data organization is crucial for ensuring its proper function.

#### 4.1.1. Required Data Organization

**Figure 4:**
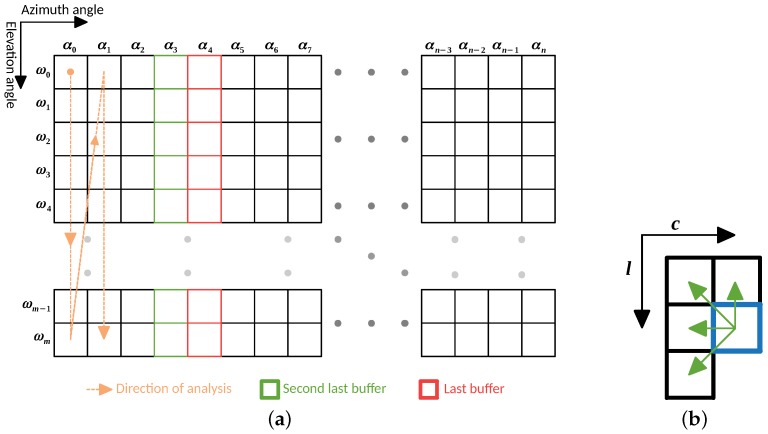
(**a**) LiDAR data organization; and (**b**) layout for neighbor comparison.

The input point cloud is treated as a 2D matrix, where each column represents an azimuth angle α, and each line an elevation angle ω. Due to time constraints, the stream of data provided by the LiDAR is assumed to be acquired (and sent) sequentially in a raster-scanning order, being used the same order for the data analysis (orange arrow in Figure 4a). During the data processing, some already processed points are kept into memory to perform a neighbor comparison based on the layout presented in Figure 4b. Those points are saved into two buffers: one of them contains the last processed points for each elevation angle, and another contains the second last processed points (red and green cells in Figure 4a, respectively). The layout chosen for neighbor comparison is the one that takes the least iterations to compare all the points in the data matrix, considering the direction of analysis.

#### 4.1.2. Adaptive Neighbor Comparison

**Figure 5:**
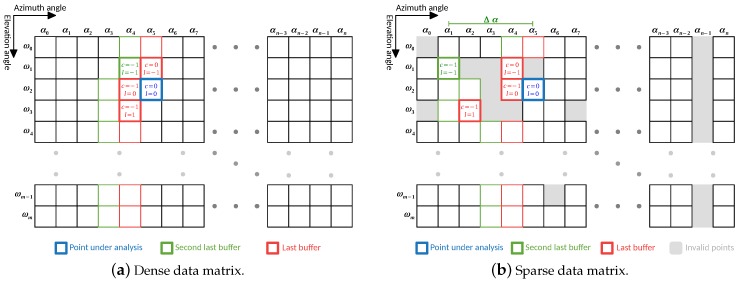
Point analysis procedure.

Whenever the input point cloud is dense, the data matrix cells are all properly filled, and the neighbor comparison is performed between contiguous cells. In Figure 5a, we show the importance of the second last points buffer (bold green cell in Figure 5b) to perform the point evaluation. In cases where the point cloud is sparse, i.e., it might have some invalid points (from invalid ranges), the layout for neighbor comparison can be adaptive to the data matrix. In Figure 5b is depicted the matrix representation of a point cloud with invalid points.

When analyzing a point, if one of its contiguous neighbors is an invalid point, the comparison can be made with a non-contiguous one. In this case, the value of Δα is evaluated and, if below a threshold, the point is compared with that neighbor, otherwise, it is assumed that the point has no neighbor for that position. Comparing Figure 5a,b is possible to notice how the layout of neighbors is adapted in the presence of invalid cells. This adaptation represents an effort to approximate a sparse data matrix to a dense analysis, reducing the effect of possible over-segmentation generated by sensor acquisition failures.

#### 4.1.3. Expected Range Calculation

**Figure 6:**
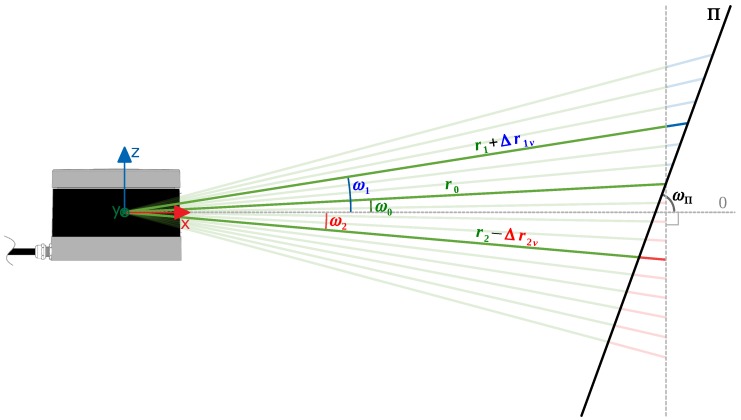
Vertical measurements of a LiDAR.

**Figure 7:**
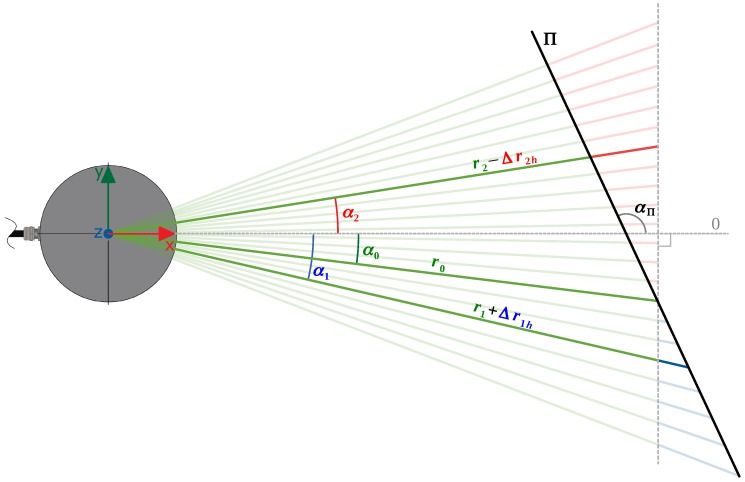
Horizontal measurements of a LiDAR.

The PL${}^{2}$DM relies on a range-based segmentation, comparing the true and expected ranges. The true range is the range actually measured by the sensor, while the expected is calculated based on the estimated plane of the cluster where the point belongs. Besides the range value, all the input points shall have azimuth and elevation angles associated. The analysis is separated into two parts: one that considers the vertical displacement (*Z*’s direction), due to differences in the elevation angle, and another related to the horizontal displacement (XY plane), associated with the azimuth angle. The vertical analysis is made by having as reference the intersection line of the plane formed by all the beams through a fixed azimuth angle, with the estimated plane (Π) of the cluster (Figure 6). The horizontal analysis is similar, but instead is considered the plane generated by some beams over a fixed elevation angle (Figure 7).

The vertical relation ($ver_{scale}$) between the reference range $r_{ref}$ and a neighbor range $r_{nb}$ is given by Equation (1):

(1) $$ver_{scale}=\frac{r_{nbver}}{r_{ref}}=\frac{\sin(\omega_{ref}-\omega_{\Pi })}{\sin(\omega_{nb}-\omega_{\Pi })}$$

The approach for the XY plane is similar to the above, and the horizontal range relation ($hor_{scale}$) is given by:

(2) $$hor_{scale}=\frac{r_{nbhor}}{r_{ref}}=\frac{\sin(\alpha_{ref}-\alpha_{\Pi })}{\sin(\alpha_{nb}-\alpha_{\Pi })}$$

Combining the vertical and horizontal relations, the expected neighbor range ($r_{nb}$) is:

(3) $$r_{nb}=hor_{scale}\cdot ver_{scale}\cdot r_{ref}$$

When performing a comparison between a point and its neighbor, if the cluster where the neighbor belongs is suitable for being approximated to a plane Π, $\omega_{\Pi }$ and $\alpha_{\Pi }$ are calculated based on its normal, otherwise, it is assumed that $\omega_{\Pi }=\pi /2$ (orthogonal to XY) and $\alpha_{\Pi }=\alpha_{ref}+\pi /2$ (perpendicular to the reference beam). After calculating them, the angular values are constrained to the interval $\omega_{\Pi },\alpha_{\Pi }\in \left[0,\pi \right]$.

An important note to this method is the fact that Equations (1) and (2) can have a null denominator, so they must be used with special care. For example, in Equation (1), this happens when $\omega_{nb}=\omega_{\Pi }$ or $\omega_{nb}=\omega_{\Pi }\pm \pi$. Even when those equalities are avoided, a very small value of the denominator will rapidly increase the value of $ver_{scale}$, which means that the relation between $\omega_{nb}$ and $\omega_{\Pi }$ needs to be limited by setting a maximum to the allowable value of $ver_{scale}$. The algorithm will consider the calculated value of $r_{nb}$ not valid if it overpasses the maximum range of the LiDAR.

Another physical limitation that is applied to this approach is the fact that the value of a range is always positive. To ensure this, $ver_{scale}$ and $hor_{scale}$ are only accepted if they are positive, so their numerator and denominator must be both positive or negative, which corresponds to, in the case of the $ver_{scale}$ value, $\omega_{ref},\omega_{nb}\in ]\omega_{\Pi },\omega_{\Pi }+\pi [$ or $\omega_{ref},\omega_{nb}\in ]\omega_{\Pi }-\pi ,\omega_{\Pi }[$, respectively. As the comparison is performed between neighbor beams, $\omega_{ref}$ and $\omega_{nb}$, and $\alpha_{ref}$ and $\alpha_{nb}$, have similar values, so these limits will likely not be respected only in cases where their values are close to $\omega_{\Pi }$ and $\alpha_{\Pi }$.

Whenever some of the presented constraints is not satisfied, the values of $\omega_{\Pi }$ and $\alpha_{\Pi }$ are set to their default, $\omega_{\Pi }=\pi /2$ and $\alpha_{\Pi }=\alpha_{ref}+\pi /2$.

#### 4.1.4. Point Clustering

There are two properties of the points that are used to create clusters. Besides the comparison between the beam true and expected ranges, it is also evaluated the relation of the normals. One of the normals is obtained directly from the previously estimated plane where the neighbor points lie, and another results from the local planar estimation. This local estimation comprises the point under analysis and the neighbors that pass the range relation constraint.

The points are first compared with their neighbors regarding the range values, using the methodology detailed in Section 4.1.3. The points may then be clustered if the relation of the true ($r_{true}$) and expected ($r_{exp}$) ranges is below a predefined threshold $\zeta_{range}$:

(4) $$\frac{\max(r_{true},r_{exp})}{\min(r_{true},r_{exp})}-1<\zeta_{range}$$

Having a valid estimated normal for the local plane, the comparison between the angles of the local and the reference (Π—associated to the neighbor’s cluster) planes is achieved by calculating the minimum angle (θ) between their normals, that is obtained from the result of their dot product:

(5) $$\theta =\arccos\left(\frac{\left|n_{\Pi_{local}}\cdot n_{\Pi }\right|}{\left|\left|n_{\Pi_{local}}\right|\right|\cdot \left|\left|n_{\Pi }\right|\right|}\right)$$

After calculating the value of θ, the points will belong to the same cluster if θ is below a predefined angular threshold ($\theta <\zeta_{angle}$). If the cluster of the neighbor point has no valid normal to represent it, the clustering is made based only in the range threshold ($\zeta_{range}$). The same happens if the reference point has no sufficient neighbors to form a plane, i.e., less than two. When the point being tested has more than one valid neighbor to associate, belonging to distinct clusters, the two clusters are merged.

When a point is associated with a cluster, the resulting representative normal is adjusted to the weighted average of it with the normal calculated for the point. Merging two clusters results in an expansion of the cluster with the lowest index, adding the points of the other cluster and averaging the representative normals, properly weighted based on the number of points.

#### 4.1.5. Cluster Classification

The generated clusters are classified as *planar*, *potential lines* and *undefined*. The *planar* clusters usually have a great number of points clustered with a small error between their expected and true ranges and θ near to zero. The clusters classified as *potential lines* are, generally, composed by a set of few points that have the same properties of the *planar*’s when compared with *potential lines* neighbors. When compared with *planar* neighbors, these kinds of clusters have a large range relation error. The type of the clusters is determined by a voting system. Whenever some of the properties mentioned above is verified, the corresponding voter is incremented. If the number of votes for a type, in relation to the number of points, is not significant, the cluster is classified as *undefined*.

### 4.2. Line Detection

**Figure 8:**
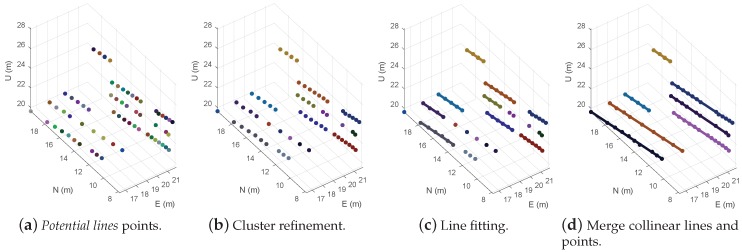
Line detection process.

The line detection algorithm is a separate thread that is triggered whenever new *potential line* points are generated. It is cause for analyzing the incoming points, refining their clustering, and for trying to fit those clusters to a straight line. From this processing stage, the points are mapped into a global frame, in order to allow the line matching between scans. The sequence of processing is illustrated in Figure 8.

#### 4.2.1. Cluster Refinement

Refining the clusters (Figure 8b) of the received points is needed to increase the quality of the line generated by the fitting step. This first clustering is based on the distance between points. For associating the points, it is followed an approach similar to the RBNN clustering [36], using a predefined $d_{max}$ as a breaking condition to stop the process. The value set for $d_{max}$ is based on the minimum expected distance between two power lines present in the environment of the inspection.

#### 4.2.2. Line Fit

The algorithm analyzes the points of the created clusters and tries to approximate them to a 3D straight line, using the eigenvalues of the unbiased covariance matrix (Figure 8c). The process of line fit is only applied to clusters with more than three points, in order to decrease the probability of having an erroneous estimation of a line.

Due to some possible gaps on the detection, some line segments shall be merged (Figure 8d). For that, the collinearity of the distinct fitted lines is evaluated, using three parameters: the angular difference, the shortest distance and the local distance.

### 4.3. Power Line Modeling

The power line modeling is the last step of the proposed algorithm. Here, the estimated line segments received from each scan are matched and grouped. Once grouped, this process tries to estimate the mathematical model of the power lines. If some power line is detected, the information about its positioning can be returned to feed the segmentation process of the next scans.

#### 4.3.1. Line Matching

The lines detected in each scan are matched and stored into sets. The new incoming lines are compared with all the last added line segments of the sets. They are then added to the set if they respect some collinearity constrains.

#### 4.3.2. Model Estimation

**Figure 9:**
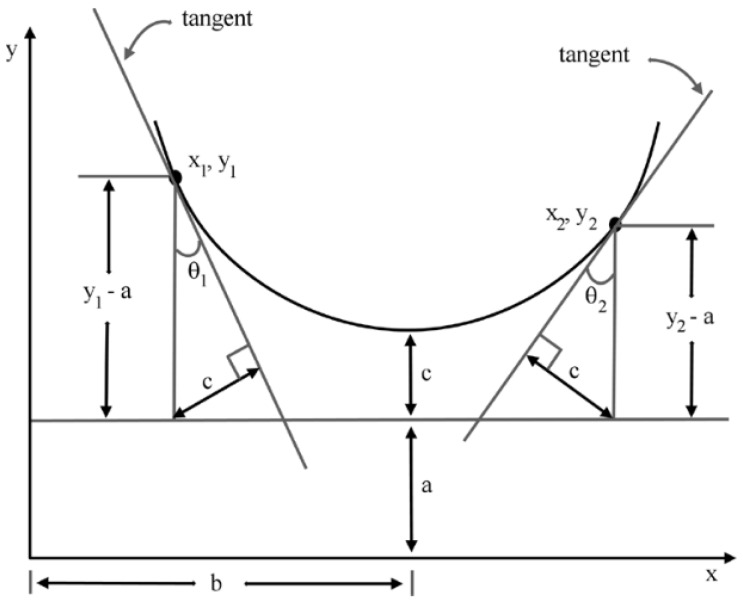
Computing catenary parameters(*a*, *b*, *c*) [59].

For online processing of the data, knowing where the lines are at each moment, is far more important than precisely matching them with a mathematical model. For this reason, the model estimation relies on the method presented in [59] (Figure 9), using only the first and last added lines of each set. The power line to be estimated is modeled by a straight line into the horizontal plane and by a catenary curve (Equation (6)) into the vertical one.

(6) $$y=a+c\cdot \cosh\left(\frac{x-b}{c}\right)$$

The values of *a*, *b* and *c* are obtained from Equation (6) and Figure 9. The estimated model is considered valid if the following constrains are respected:

- $\theta_{first}\neq \theta_{last}$;
- $a<z_{first}$ and $a<z_{last}$;
- c>0;
- $\frac{z_{first}-a}{c}\geq 1$.

Due to the use of the catenary in a 3D domain, *z* corresponds to *y* in the Equation (6) and Figure 9.

#### 4.3.3. Parameter Return

The algorithm returns the power line models, if any, associated to a bounding box and the direction (sensor’s azimuth) of the last detected lines. This information can be used to feed the segmentation algorithm, refining its output for the subsequent scans.

## 5. Results

**Figure 10:**
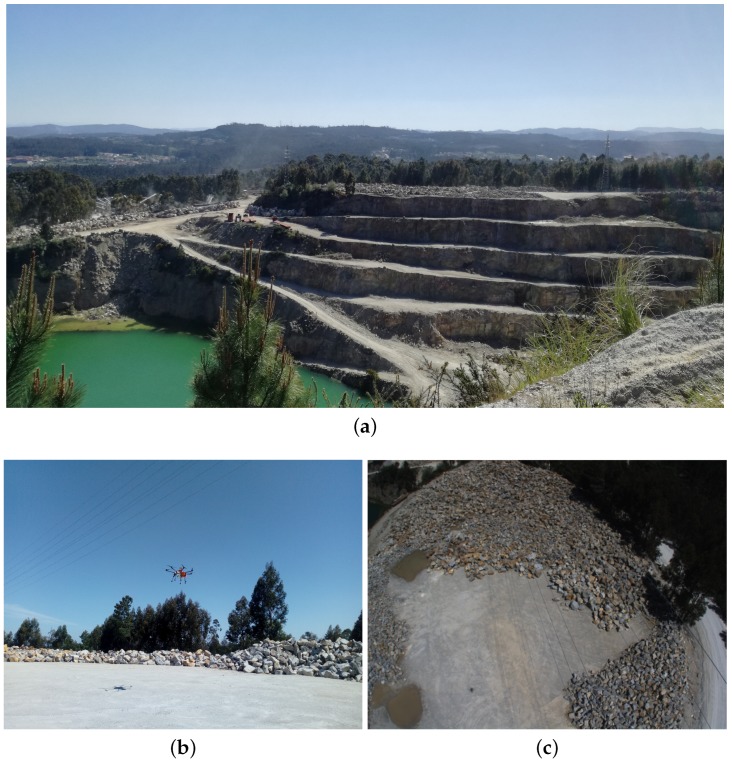
(**a**) Malaposta quarry overview; (**b**) STORK UAV mapping the stockpile; and (**c**) STORK UAV onboard images.

The dataset was performed during a mapping survey of a rock stockpile in the Malaposta quarry (Figure 10), in Santa Maria da Feira, Aveiro, Portugal, using the UAV STORK [66] with a Velodyne VLP-16 [67]. Above the mapped stockpile, there was a span of 6 power lines and a guard cable, which made this dataset suitable for testing the PL${}^{2}$DM.

### 5.1. Experimental Setup

**Figure 11:**
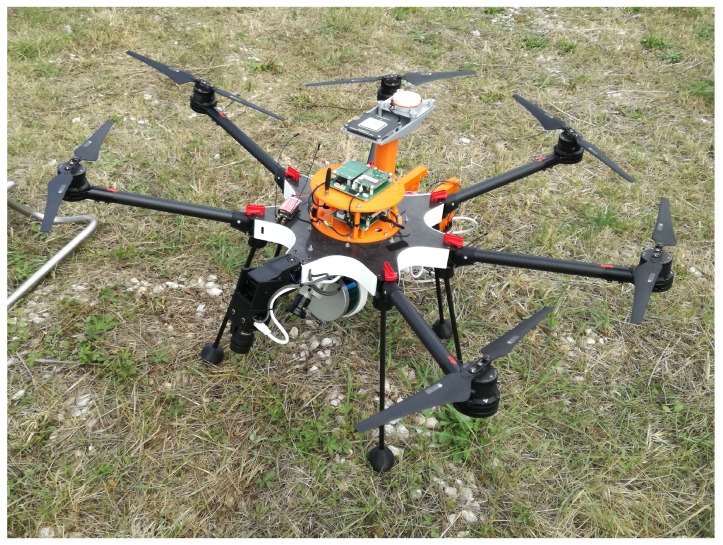
STORK UAV.

The multirotor UAV STORK [66] (Figure 11) is a custom hexacopter designed to achieve both efficiency and versatility. Its primary application is the power assets inspection. Nonetheless, it has also been used in the first trials of the SpilLess project [3,68], and in several precise mapping surveys. This range of applications can be attained by means of an adaptive payload methodology: using the same frame, payload sensors can be easily replaced by others that provide a different type of data.

This UAV is capable of navigating in both manual and autonomous modes, having also the ability to perform some autonomous maneuvers, like takeoff, landing or the inspection of a structure, using the onboard sensors. In its current state, STORK UAV is low-level controlled by a customized autopilot (INESC TEC Autopilot) and has an onboard computer that is responsible for controlling the UAV at a higher level (ODROID-XU4, running Ubuntu 16.04 LTS and ROS Kinetic Kame).

For navigation, this UAV has two IMUs and two GNSS receivers. Besides the default low-cost IMU sensors, the autopilot can also use the STIM300, that is a high-performance IMU. Similarly, the single-band GNSS receiver Ublox NEO-M8T is the low-cost alternative of ComNav K501G, a dual-band receiver that supports onboard Real-Time Kinematic (RTK) positioning [69].

Although the low-cost combination of sensors generally fulfills the applications requirements, others like precise mapping surveys imply the use of highly accurate sensors.

The STORK UAV can percept the surrounding environment by using two visual cameras (a fixed Teledyne Dalsa G3-GC10-C2050, pointing 45 degrees forward-down, and a FLIR PointGrey CM3-U3-13S2C-CS, attached to a gimbal), a LiDAR sensor (Velodyne VLP-16) and, in some applications, a thermographic camera (FLIR A65). The data provided by these sensors can be used as an input for processing algorithms that will be used by the navigation layer, or to create other outputs, like 3D models of a structure.

As the main objective of the flight was the mapping of the stockpile, the UAV had the Velodyne sensor mounted under its frame, pointing downwards, with a negative 75 degrees pitch rotation. This kind of configuration allows the LiDAR sensor to percept only the environment below and sideways with respect to the UAV position. This means that everything that is immediately above, in front or in the back of the UAV cannot be detected.

### 5.2. Dataset

**Figure 12:**
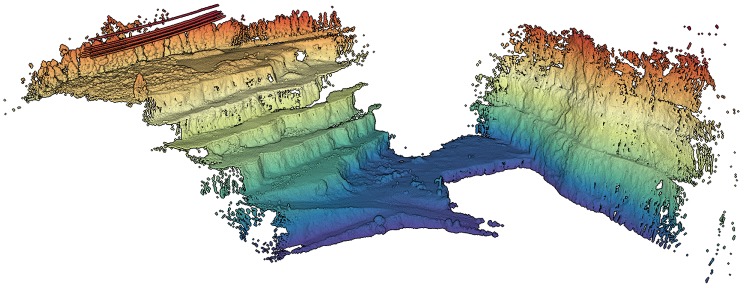
Complete point cloud of the Malaposta quarry mapping survey.

During the mapping survey in the Malaposta quarry, the UAV has performed three flights. Combining all the collected data, we obtained a point cloud with nearly 110 millions of points (http://lsa.isep.ipp.pt/~adias/cloud/minas/malaposta_pointcloud2.html) (Figure 12). The main objective of this survey was the precise mapping of part of the quarry, which means that the UAV was operating with the high-performance IMU STIM300 and the GNSS receiver ComNav K501G. Therefore, the UAV pose was estimated by fusing their measurements. To synchronize the timestamps of all subsystems was used the PPS signal. This synchronization allowed the post-processing of the point cloud, associating each LiDAR point to a timestamp and then to a pose of the UAV, estimated at a rate of 1 kHz.

The PL${}^{2}$DM was tested using only one of the three available flight datasets, in which the power lines were detected. For the online processing of the point cloud, a single timestamp was associated to the whole scan, instead of time labelling each point individually. This means that the precision of the real-time global mapping of the power lines may be affected.

### 5.3. Segmentation and Point Classification

**Figure 13:**
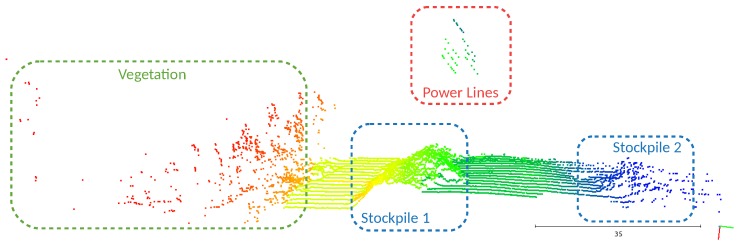
Object identification in the point cloud.

**Figure 14:**
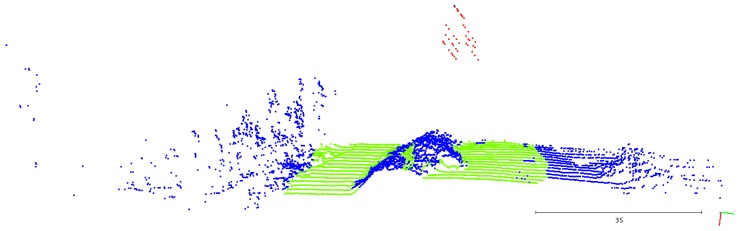
Segmented point cloud from one scan, colored by point type. Green corresponds to *planar*, red to *potential lines* and blue to *undefined*.

For a better understanding of the resultant clusters, in Figure 13, we identified the detected structures. Having the point cloud associated with the structures, it becomes evident that clusters’ breakpoints occur mainly in the presence of the power lines, stockpiles, and vegetation. From the resultant clusters, the algorithm classifies the points based on a voting system that evaluates the relations between a point and its neighbors (Figure 14).

**Figure 15:**
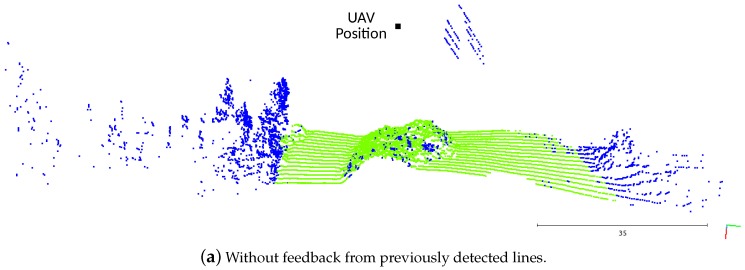

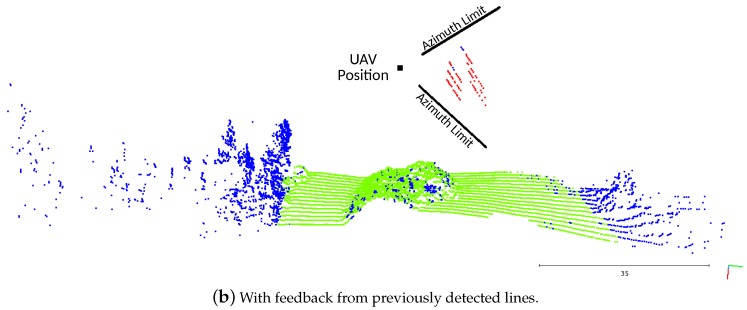
Effect of the feedback about previously detected lines.

In Figure 15a, the algorithm was running without getting any feedback about the positioning of the lines from the previous scans. As they were detected by the LiDAR without any structure behind them, the voter is not capable of distinguishing them from the vegetation or outliers, attributing the *undefined* classification. Adding the information feedback (Figure 15b), the point classification task had a better performance, correctly labeling almost all the power line points as *potential lines*.

### 5.4. Power Line Modeling

**Figure 16:**
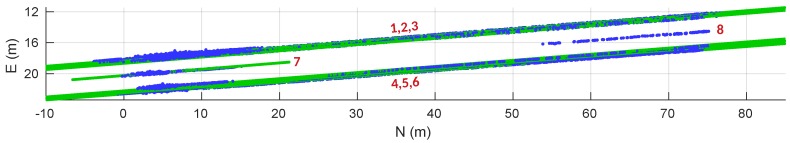
Horizontal analysis of estimated power line models (green) and line segments centers (blue).

**Figure 17:**
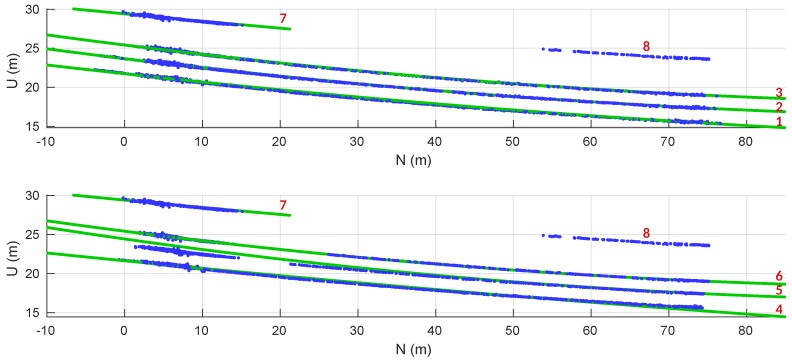
Vertical analysis of estimated power lines.

The power line modeling was based in the direction and center points of all the line segments associated to the same cluster. In Figure 16 and Figure 17, the valid power line models obtained are displayed in green and the line segment centers in blue. The red numbers correspond to a labeling of the detected power lines to match the results presented in Table 1.

From Figure 16 we can perceive that the power line cluster 8 has no model associated. The algorithm was not capable of reaching a valid model due to the straightness of the segment.

The power lines were modeled as a straight line in the horizontal, with a direction obtained from the average of the correspondent line segments direction. Their vertical model was an approximation to a catenary curve. The catenary parameters used are the ones that, at each moment, minimize the vertical error between the model and the line segments centers.

The power line clusters 1, 2 and 3 have a vertical model that is better adjusted to their data points when compared to the clusters 4, 5 and 6. This happened because the power lines 1, 2 and 3 were continuously seen from the moment when they were first detected, unlike the others. The clusters 4, 5 and 6 were only partially detected in the first part of the flight. The rest of their line segments were sequentially merged in an inverse way, when the UAV was returning to the initial point.

In Table 1 are listed the fitting errors of the estimated models. Apart from line 5, with an absolute vertical mean error of 0.49 m, all the other lines have an estimation with an average absolute error smaller than 0.07 m. In the horizontal fitting, all the mean errors are placed below the 0.14 m.

**Figure 18:**
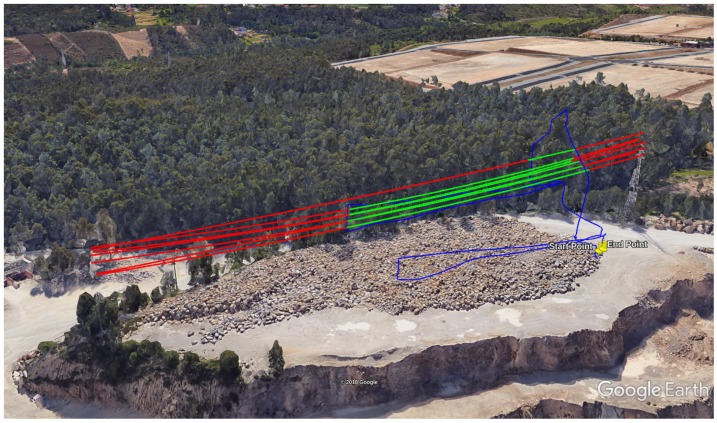
Power line models represented in Google Earth. Estimated models, in green, with their expansion in space, in red. UAV trajectory in blue.

Although some of the models might not be precisely fitted, their expansion into space allows a prediction of where the power lines are globally placed. Exporting the values of the power line parameters obtained, higher-level algorithms can evaluate the reliability of the models and expand them accordingly. In Figure 18, we projected the obtained models in Google Earth. It can be seen that the lines lie between the two associated pylons, connecting to them when the models are expanded. This allows us to validate the obtained models.

### 5.5. Performance Evaluation

#### 5.5.1. Line Points Classification

The LiDAR sensor was able to measure almost 35 millions of points during the dataset used from one flight. From those, 86,887 points corresponded to power lines. The performance evaluation of the PL${}^{2}$DM considers only the points correspondent to power lines. Following the strategy in [64], the completeness ($C_{m}$) and correctness ($C_{r}$) are given by:

(7) $$C_{m}=\frac{TP}{TP+FN}$$

(8) $$C_{r}=\frac{TP}{TP+FP}$$

Being TP the true positive results, i.e., the points correctly labeled as *potential lines*, FN the false negatives (true power line points not classified as *potential lines*), and FP the false positives or the points wrongly labeled as *potential lines*.

Regarding the outputs of the segmentation step, the algorithm has classified 69,914 points as *potential lines*. However, some FP points were generated (1038 points). For this case, the number of the manually labeled power line points corresponds to the TP+FN value. The values of correctness ($C_{r\_class}$) and completeness ($C_{m\_class}$) of the point classification are 79.27% and 98.52%, respectively.

During the line fitting process, some of the *potential lines* points are not considered. Thus, its completeness ($C_{m\_fit}$) and correctness ($C_{r\_fit}$) can be evaluated against the received points. Here, the value of TP+FN corresponds to 68,876 points. For this case, the generated power line points has no FN points and 66,624 TP ones. Here, the value of $C_{m\_fit}$ was 96.73%, and 100% of $C_{r\_fit}$.

In order to evaluate the advantage of using the power line model feedback for the segmentation, we also evaluated the correctness and completeness ($C_{r\_no\_feed}$ and $C_{m\_no\_feed}$) with no feedback. The obtained values were 31.18% for $C_{m\_no\_feed}$ and 96.36% for $C_{r\_no\_feed}$.

Table 2 lists all the calculated values, being noticeable an increase of nearly 48% in the completeness when using the feedback of the previously estimated models.

#### 5.5.2. Processing Time

During the real-time dataset execution, Velodyne VLP-16 was providing data at an approximated rate of 10 Hz and working in the single *Strongest* return mode. With this information, the algorithm will be able to run in real-time if it processes the data and generates outputs in less than 100 ms.

**Figure 19:**
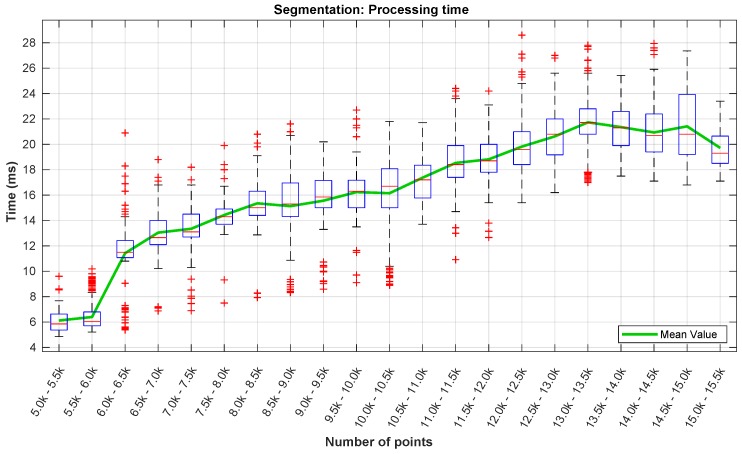
Processing time of the segmentation step.

**Figure 20:**
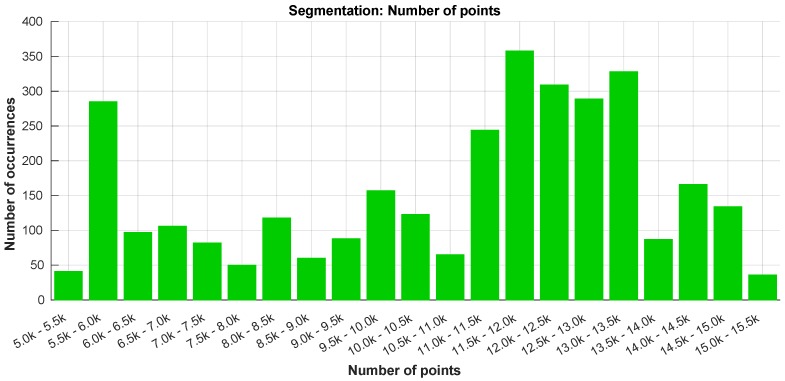
Number of points occurrences in the segmentation step.

Due to the quantity of data involved, the segmentation step is the most crucial in terms of the overall processing time of the algorithm. In Figure 19, we present the processing time of the segmentation layer in terms of the number of data points. As the LiDAR was mounted below the UAV’s frame, almost half of its data points is neither valid nor useful. Returning a maximum of ∼30,000 points per scan, with this mounting configuration, this means that, from the outset, having a number of points above 15,000 in one scan is almost impossible. There were no occurrences of scans with more than 15,500 points per scan in the dataset (Figure 19 and Figure 20).

Regarding the processing time (Figure 19), it is visible an increase with the number of data points. This was expected due to the fact of the segmentation algorithm’s loop nature. The highest processing time registered was around 29 ms, while the highest mean time (in green) is below 22 ms. For the same number of points, the variation of the processing time values is closely related with the number of neighbors associated to each point.

In Figure 20 can be observed that the LiDAR was returning a number of points between 11,000 and 13,500 in most of the time. The peak registered in the 5–6000 points is related to the time while the UAV was on the ground.

**Figure 21:**
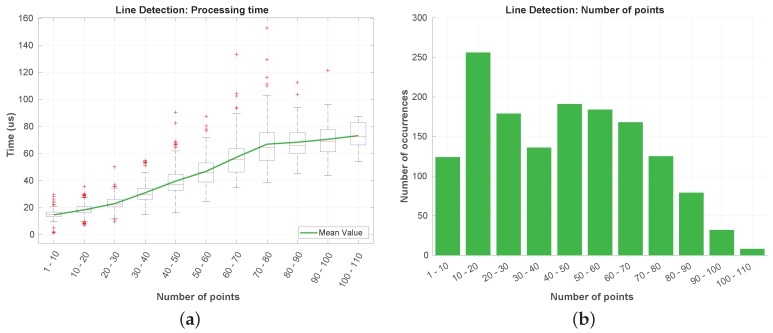
Processing time (**a**) and number of points occurrences (**b**) in the line detection.

The results obtained for the line detection step are depicted in Figure 21. The number of points that reaches this step is typically under a hundred (Figure 21b), being noticeable an increase of the processing time with the number of incoming points. In Figure 21a can be seen that the processing time is almost negligible when compared to the segmentation processing times. The highest time registered was under the 160 μs.

**Figure 22:**
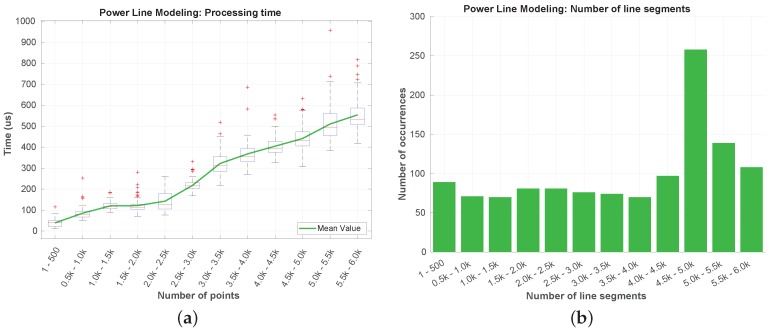
Processing time (**a**) and number of line segments (**b**) for power line modeling.

In the last step of the algorithm, the power line modeling, were obtained and the results exposed in Figure 22. After being associated, the line segments of the power line clusters are not removed. This leads to a growing processing time over the flight duration. In Figure 22a, we verify that, during all the dataset, the processing time has never overpassed the 1 ms. This constant increase in the time value is related to the evaluation of the vertical error whenever some new valid parameters *a*, *b*, and *c* are found. In Figure 22b, we show a dominance in the occurrence of a number of line segments between 4500 and 5000. This may refer to a period of the flight where few new line segments were added to the power line models at each iteration. In the rest of the flight, the line segments number had an almost constant growth.

**Figure 23:**
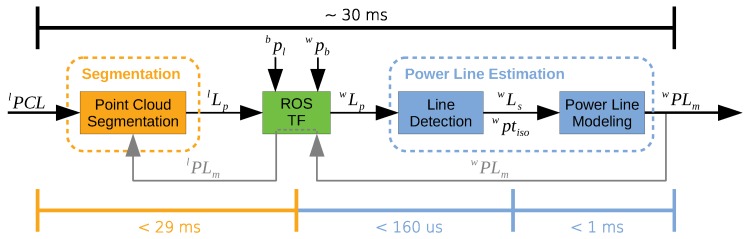
Total processing times of the PL${}^{2}$DM.

Figure 23 resumes the obtained processing times values. The maximum processing time for segmenting the point cloud was ∼29 ms, less than ∼160 μs for the line fitting, and under 1 ms for the power line modeling. From there, it can be concluded that the algorithm is capable of processing more than 500,000 points each second and still fulfill the real-time requirement.

The processing times were obtained in a computer with an Intel Core i7 4720HQ processor and 8 GB of RAM. The processing was made in the Ubuntu 18.04 LTS operating system, inside the ROS Melodic Morenia framework.

### 5.6. Discussion

Some considerations regarding the obtained results were already made along Section 5. The PL${}^{2}$DM is capable of successfully segment a point cloud and classify correctly almost all the points that corresponded to power lines. In this classification process, using the feedback of the already estimated power line models for the next scans turned out to be very useful. When using the feedback, the completeness value ($C_{m}$) had an increase of nearly 48%, which means that the algorithm has correctly labelled almost 80% of the available power line points and used them for modeling the power lines.

Even not considering 20% of the available power lins points, the obtained power line models were fitted with a low error. For the vertical fitting, the maximum mean absolute error was 0.49 m, but the second largest was only 0.07 m. In the horizontal, all the errors were below 0.14 m. When applying the output of the algorithm to an obstacle detection layer, these errors are reasonable: setting up a safe distance threshold to the power lines will always avoid a collision. Adding to that, the prediction of the power lines model extension can help in path planning tasks, either for collision avoidance or for line following maneuvers.

**Figure 24:**
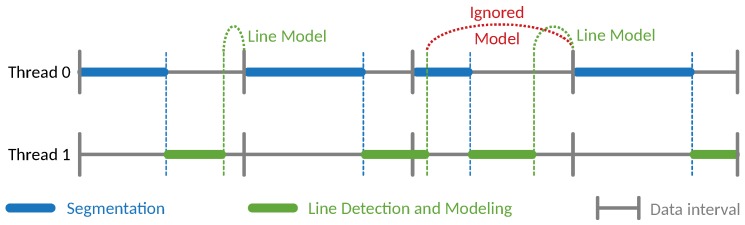
Effect of slow processing.

Considering the processing time, the algorithm has been shown to be able to run in real-time for processing up to half a million points per second. However, when using the proposed algorithm in a situation where the processing time is close to the available time, there are some considerations that must be observed. The algorithm was designed for having a parallel processing of the segmentation and line detection and modeling (Figure 24). Whenever the segmentation is performed, if some *potential line* points are found, the other thread is triggered (blue dashed line in Figure 24). From the line detection and modeling thread, we find the line models to the next scan to aid the segmentation (green dashed line in Figure 24).

If the combined time of the segmentation and the line detecting is greater than the available time (imposed by the sensor’s data rate), the data may desynchronize. The next scan will use the same line model as the previous and the new line model can be either ignored or delayed for the subsequent scans. In the case depicted at Figure 24, in red, the generated line model is ignored by the next scan due to the existence of newer data. However, if no new model is generated, is used the one obtained from the two (or more) previous scans data.

During a long flight or in a scenario with several power lines, the constant increase of the processing time corresponding to the power line modeling might cause the slow processing effect (Figure 24). To overcome this, some down-sampling strategy of the line segments can be applied when the confidence in the estimated model is high

## 6. Conclusions and Future Work

This paper has focused on the development of an algorithm capable of detecting and modeling surrounding power lines in real-time, the PL${}^{2}$DM. The data is provided by a LiDAR sensor mounted in a multirotor UAV. Its outputs and performance were evaluated using a dataset containing several types of structures, acquired during a mapping survey in the Malaposta quarry. The algorithm was able to segment an input point cloud, detect power lines points and generate line segments, that are merged to construct the final power line model. The whole process has shown to be suitable for being applied in tasks with real-time requirements. Therefore, this work contributes to the existing literature by addressing the lack of solutions for detecting and modeling power lines in real-time, dealing with a large amount of data at a high rate provided by a 3D LiDAR.

In the classification of power line points, the algorithm fails to label some of them as *potential lines*. This is due to the difficulty in discerning between power lines and vegetation points, labeling them as *undefined*. Nonetheless, the power line modeling is still reached with low associated error in most of the cases. The knowledge of those line models for the next scans segmentation has turned out to be very useful in helping the point classification, increasing in 48% the number of power line points used for modeling.

To the authors’ knowledge, the contribution to the segmentation’s methodology is derived from a novel approach based on planar properties of the structures. In the line detection and modeling steps, there is also a contribution to the existent methods. It applies a methodology that does not rely on the HT to detect lines nor assumes a flight direction parallel to the power lines. Instead, it uses collinearity properties and accepts lines from any direction. Other advantages of this algorithm is being suitable for both sparse and dense point clouds, and independent on the type of vehicle that carries the sensor, as all of the analysis is made with respect to the LiDAR.

Although the concept of the algorithm was validated with the available dataset, it still needs to be validated with others with different conditions. One of those may be the detection of multiple spans of a power line set, or even in scenarios with multiple distinct lines. Having different types of backgrounds (vegetation, rocks, buildings, etc.) or lines with various slopes are other interesting conditions to validate the method. For trying to improve and refine the point classification, the use of the LiDAR in dual-return mode can be tested. However, the algorithm would need adaptations to consider the double point existence.

When migrating the algorithm to new processing units, the running time needs to be re-evaluated to either consider or not the effect of slow processing, depicted in Figure 24. An alternative version of the algorithm, implemented in a Graphics Processing Unit (GPU), can be advantageous to the performance of the algorithm, especially in the segmentation step. The scan could be divided into several parts and analyzed in parallel, being then merged by processing the contiguous limits of each part.

On top of this work, several higher-level algorithms can be developed using the data provided. The power line model is useful for obstacle detection, collision avoidance, or path planning methods, and like line following. At the height of the power line, the *undefined* points usually correspond to vegetation. Associating this with the obtained models allows the application of vegetation clearance anomalies detectors. The horizontal *planar* structures are useful to detect possible safe landing spots for a UAV.

## Figures and Tables

**Table 1:** Power line estimated model errors.

	Error			
	Horizontal		Vertical	
Line	μ(m)	$\sigma^{2}$(m${}^{2}$)	μ(m)	$\sigma^{2}$(m${}^{2}$)
1	0.12	0.0100	0.07	0.0080
2	0.09	0.0111	0.04	0.0036
3	**0.14**	0.0151	0.05	0.0046
4	0.07	0.0070	0.07	0.0239
5	0.12	0.0142	**0.49**	0.0726
6	0.12	0.0134	0.04	0.0035
7	0.06	0.0042	0.05	0.0052
8	0.03	0.0009	-	-

**Table 2:** Point classification performance.

Classification				Line Fit	
$C_{m\_\mathrm{no}\_\mathrm{feed}}$	$C_{r\_\mathrm{no}\_\mathrm{feed}}$	$C_{m\_\mathrm{class}}$	$C_{r\_\mathrm{class}}$	$C_{m\_\mathrm{fit}}$	$C_{r\_\mathrm{fit}}$
31.18%	96.36%	79.27%	98.52%	96.73%	100%

## Abbreviations

The following abbreviations are used in this manuscript:

CLF	Compass Line Filter
e-RBNN	Ellipsoid model based RBNN
ED	Euclidean Distance
ESCS	Extruded Surface of Cross Section
GNSS	Global Navigation Satellite System
GP-INSAC	Gaussian Process INcremental SAmple Consensus
GPF	Ground Plane Fitting
GPU	Graphics Processing Unit
HT	Hough Transform
IMU	Inertial Measurement Unit
LiDAR	Light Detection And Ranging
NN	Nearest Neighbor
PL${}^{2}$DM	Power Line LiDAR-based Detection and Modeling
PPS	Pulse Per Second
RANSAC	RANdom SAmple Consensus
RBNN	Radially Bounded Nearest Neighbors
ROS	Robotic Operating System
RTK	Real-Time Kinematic
SLR	Scan Line Run
SVM	Support Vector Machine
UAV	Unmanned Aerial Vehicle
VPLD	Voxel-based Piece-wise Line Detector
